# Prevalence and risk factors of stress urinary incontinence in a 2023 Japanese community health survey ‐ differences between males and females

**DOI:** 10.1002/bco2.70004

**Published:** 2025-02-17

**Authors:** Nobuhiro Haga, Mikako Yoshida, Takahiko Mitsui, Noritoshi Sekido, Naoya Masumori, Kenji Omae, Motoaki Saito, Yasue Kubota, Ryuji Sakakibara, Satoru Takahashi

**Affiliations:** ^1^ Epidemiological Survey Executive Committee the Japanese Continence Society Japan; ^2^ Department of Urology Fukuoka University Faculty of Medicine Fukuoka Japan; ^3^ Department of Women's Health Nursing & Midwifery Tohoku University Graduate School of Medicine Miyagi Japan; ^4^ Department of Urology University of Yamanashi Graduate School of Medical Sciences Japan; ^5^ Department of Urology Toho University Ohashi Medical Center Japan; ^6^ Department of Urology Sapporo Medical University School of Medicine Japan; ^7^ Department of Innovative Research and Education for Clinicians and Trainees (DiRECT) Fukushima Medical University Hospital Japan; ^8^ Department of Pharmacology, Kochi Medical School Kochi University Japan; ^9^ Department of Advanced Medical Nursing Nagoya City University Graduate School of Nursing Japan; ^10^ Neurology Clinic Tsudanuma and Dowakai Chiba Hospital Japan; ^11^ Department of Urology Nihon University School of Medicine Japan

**Keywords:** epidemiology, faecal incontinence, female, male, stress urinary incontinence

## Abstract

**Objectives:**

The aim of the present epidemiological study was to evaluate the sex‐related prevalence of stress urinary incontinence (SUI) and the associated factors using data from the 2023 Japan Community Health Survey.

**Methods:**

We investigated 3097 males and 3056 females aged 20–99 years. All participants answered web‐based questionnaires on their health status and lower urinary tract symptoms. Data on the frequency of SUI, comorbidities and health‐related behaviour were extracted. The Cochran‐Armitage trend test was used to evaluate the trend between the prevalence of SUI and age. Multivariate analysis was performed using logistic regression analysis to identify factors associated with SUI.

**Results:**

SUI was consistently observed in about 10% of individuals in their 20s and 30s, including in males. There were no age‐related differences in the prevalence of SUI in males (P = 0.55). In females, the prevalence of SUI statistically significantly increased with age (P < 0.0001). The frequency of SUI was, however, low in both sexes. Drinking habits (OR, 1.43; 95% CI, 1.10–1.87) and frequent spicy food intake (OR, 1.55; 95% CI, 1.19–2.01) were associated with SUI only in males. Age (OR, 1.36; 95% CI, 1.13–1.62), BMI (OR, 1.87; 95% CI, 1.50–2.32) and history of vaginal delivery (OR, 2.15; 95% CI, 1.77–2.63) were only associated with SUI in females.

**Conclusions:**

Although the frequency of SUI was low in both sexes, the correlation between the prevalence of SUI and age was different between both sexes. Female SUI might involve weakness of the pelvic floor muscle, while male SUI might be affected by health‐related behaviours.

## 1 INTRODUCTION

To date, although there have been many epidemiological studies on stress urinary incontinence (SUI) in females, only a few epidemiological studies on SUI have been conducted in males.^1^, ^2^, ^3^, ^4^ While SUI was reportedly observed in about 35% of women who underwent CrossFit® training,^5^ it was not observed in men who underwent the same training.^2^ Thus, although there might be sex differences in the prevalence of SUI, they have not been clarified to date.

The causes of SUI in males and females are reportedly different. Female SUI is induced by sphincter deficiency, decreased elasticity of the urethral wall, weakening of the sealing effect of the urethral mucosa and impaired pelvic floor support induced by vaginal delivery and obesity.^6^, ^7^ Urethral hypermobility is also, reportedly, involved in female SUI.^8^ The causes and associated factors involved in male SUI, on the other hand, are reportedly surgery for benign prostatic obstruction, neurogenic conditions, pelvic surgery, radical prostatectomy, etc.^9^ In radical prostatectomy, in particular, changes in the anatomy of the pelvic floor and bladder neck, as well as neurological insult following prostatectomy, might be involved in male SUI. Thus, the pathophysiology of male SUI is suggested to involve sphincter deficiency and anatomical changes in the pelvis.^9^ Since there are probably sex differences in the pathophysiology of SUI, it is possible that the factors associated with SUI might differ between the two sexes. However, the sex differences in the factors related to SUI have not yet been clarified, especially in the same cohort.

In 2003, the Japanese Continence Society conducted an epidemiological survey regarding lower urinary tract symptoms (LUTS) in Japan.^10^, ^11^ Twenty years after that previous epidemiological survey, another web‐based cross‐sectional study was conducted, named the JaCS 2023 (Japan Community Health Survey in 2023) study, to survey the prevalence and characteristics of LUTS in Japan.

The aim of the present epidemiological study was to evaluate the sex‐related prevalence of SUI and its associated factors using data acquired from the JaCS 2023 study.

## 2 MATERIALS AND METHODS

### 2.1 Study design

In the present study, the data of 6210 Japanese men and women aged 20–99 years, from among the participants in the JaCS 2023 study, were collected by an online research company (Macromill) from May 2023 to June 2023. To recruit respondents, e‐mails were sent by Macromill to potential participants of this survey, who were selected by probability sampling based on the population composition of the October 2022 National Census of the Statistics Bureau, Ministry of Internal Affairs and Communications, among each age group from 20 to 99 years old. The survey participants were divided based on sex, age, region, etc. (https://www.stat.go.jp/data/jinsui/2022np/index.html).

The participants were recruited by Macromill from among preregistered men and women. The inclusion criteria in the present study were: males and females 20–99 years of age, ability to understand Japanese, ability to answer each question via the website and provision of informed consent for study participation on the website.

Exclusion criteria in the present study were: participants who gave incomplete replies, those whose BMI values ranked in the top 0.5% (BMI>36.5 kg/m^2^), and participants whose BMI values ranked in the bottom 0.5% (BMI < 15.0 kg/m^2^), as extreme outliers. Since extreme obesity is uncommon in Japan, these BMI values were added in the exclusion criteria since we could not completely exclude the possibility of inappropriate answers for study subjects who reported extremely high or low BMI values. This research was performed in accordance with the guidelines of the Declaration of Helsinki and was approved by the ethics committee of Nihon University, Tokyo, Japan (approval number 2023–4).

The primary objective was to investigate the sex‐related prevalence of SUI per age decade. The secondary objective was to investigate factors related to SUI.

### 2.2 Data extraction

The contents of the survey questionnaire fundamentally adhered to the questionnaire published 20 years ago.^10^ However, several new items were incorporated, including questions addressing post‐void incontinence or postmicturition leakage and straining during urination, either at the beginning or throughout the process.^12^, ^13^, ^14^ Briefly, the questionnaire covered aspects such as demographic information, LUTS, daily life activities and healthcare‐seeking behaviour. Questions related to LUTS and daily life referred specifically to experiences during the preceding month. Demographic details included age, sex, parity (for women), functional ability, overall health status and any past or current comorbidities.

The LUTS assessed included storage symptoms (daytime urinary frequency, nocturia, urgency, urgency urinary incontinence and stress urinary incontinence), voiding symptoms (hesitancy, slow stream, intermittency and straining), post‐void symptoms (feeling of incomplete bladder emptying and post‐void incontinence or postmicturition leakage) and bladder pain. Symptom descriptions adhered to the terminology standards set by the International Continence Society. Responses to daytime urinary frequency and nocturia questions were recorded as actual urination counts, while other symptoms were evaluated using a frequency scale: none, less than once per week, once or more per week, roughly once daily, several times daily or always.

The main questions regarding SUI were as follows: “During the past month, did you ever leak urine when you coughed or moved around? If yes, how often?”. In addition, the frequency of SUI was divided into five grades, as follows: Grade 1: SUI is observed less than once per week; Grade 2: SUI is observed once or more than once per week; Grade 3: SUI is observed once per day; Grade 4: SUI is observed several times per day; and Grade 5: SUI is always observed through the day.

Additionally, to investigate the association between SUI and the participants' characteristics, we used the following information provided by the participants. Age and body mass index (BMI) were used as basic data. Hypertension, dyslipidemia, diabetes mellitus, heart failure, ischemic heart disease, cerebral stroke, spinal cord disorders and neurological disorders were assessed as comorbidities. Additionally, history of vaginal delivery was assessed as a comorbidity in females, and benign prostatic hyperplasia (BPH) and erectile dysfunction (ED) were assessed as comorbidities in males. ED was evaluated by the erection hardness score,^15^ with participants determined as Grade 3 or less being judged as having ED. Regarding health‐related behaviours, drinking habit, history of smoking, frequent intake of caffeine and frequent spice intake were newly assessed in the present survey. The presence of a drinking habit was determined based on the frequency of alcohol consumption Additionally, faecal incontinence and constipation, determined based on self‐reporting, were evaluated in the present survey to assess the participants' health status.

### 2.3 Statistical analyses

Continuous data are presented as the number (%) and mean ± standard deviation, and their differences between men and women were tested by the unpaired t‐test. The Cochran‐Armitage trend test was used to evaluate the trend between the prevalence of SUI and age in both males and females.

Multivariate analysis was performed using logistic regression analysis by the forced entry method to identify factors associated with SUI, regardless of the results of univariate analyses investigating the potential differences in baseline characteristics between men and women. For multivariate analyses, patients' basic data, comorbidities, health‐related behaviours and health status were selected as independent variables, as were vaginal delivery in females, and BPH and ED in males. These factors were chosen based on their clinical relevance and since they were also used in previous studies.^16^, ^17^

For logistic regression analysis, participants were divided into high‐BMI and low‐BMI groups based on a value of 25 kg/m^2^, which is the cut‐off value for diagnosing obesity in Japan.^18^ Additionally, participants were divided into old‐ and young‐age groups based on a cut‐off value of 60 years, according to the research of the National Health and Nutrition Examination Survey.^19^ Statistical significance was set at a p‐value of <0.05. Statistical analyses were performed using JMP version 11.0 software (100 SAS Campus Drive, Cary, NC, USA).

## 3 RESULTS

After excluding 57 participants due to the BMI criteria in the present study, 6153 subjects were investigated in the present analyses. They included 3097 participants in the male cohort, and 3056 participants in the female cohort (Table 1). Regarding basic data, males were significantly younger than the females in this study (P = 0.0001), and their mean BMI was significantly higher (P < 0.0001). Regarding comorbidities, hypertension (P < 0.0001), diabetes mellitus (P < 0.0001), heart failure (P = 0.01), ischemic heart disease (P < 0.0001) and cerebral stroke (P = 0.0005) occurred significantly more often in men. Regarding health‐related behaviour, intake of spice (P < 0.0001), caffeine (P < 0.0001), drinking habit (P < 0.0001) and smoking history (P < 0.0001) were significantly more frequent in males. Constipation was significantly less frequent in males (P < 0.0001) (Table 1).

**TABLE 1:** Baseline characteristic both in male and female.

Characteristics	Male	Female	p
n	3097	3056	
Age, in years, mean ±SD	53.3 ± 17.9	55.1 ± 18.6	0.0001
BMI, kg/m^2^ mean ± SD	23.3 ± 3.4	21.5 ± 3.5	<0.0001
Hypertension, n (%)	787 (25.4%)	538 (17.6%)	<0.0001
Dyslipidemia, n (%)	380 (12.3%)	360 (11.8%)	0.5569
Diabetes mellitus, n (%)	321 (10.4%)	137 (4.5%)	<0.0001
Heart failure, n (%)	83 (2.7%)	53 (1.7%)	0.0119
Ischemic heart disease, n (%)	130 (4.2%)	63 (2.1%)	<0.0001
Cerebral stroke, n (%)	93 (3.0%)	58 (1.9%)	0.0051
Spinal cord disorder, n (%)	83 (2.7%)	86 (2.8%)	0.7557
Neurological disorder, n (%)	59 (1.9%)	45 (1.5%)	0.1994
Faecal incontinence, n (%)	83 (2.7%)	70 (2.3%)	0.3679
Constipation, n (%)	266 (8.6%)	425 (13.9%)	<0.0001
Frequent spicy food intake, n (%)	1040 (33.6%)	766 (25.1%)	<0.0001
Frequent caffeine intake, n (%)	2178 (70.3%)	1988 (65.1%)	<0.0001
Drinking habit, n (%)	1212 (39.1%)	550 (18.0%)	<0.0001
History of smoking, n (%)	1877 (60.6%)	818 (26.8%)	<0.0001
Benign prostatic hyperplasia (%)	231 (7.5%)	‐	‐
Erectile dysfunction, n (%)	1304 (42.1%)	‐	‐
Vaginal delivery, n (%)	‐	1906 (62.4%)	‐

**FIGURE 1:**
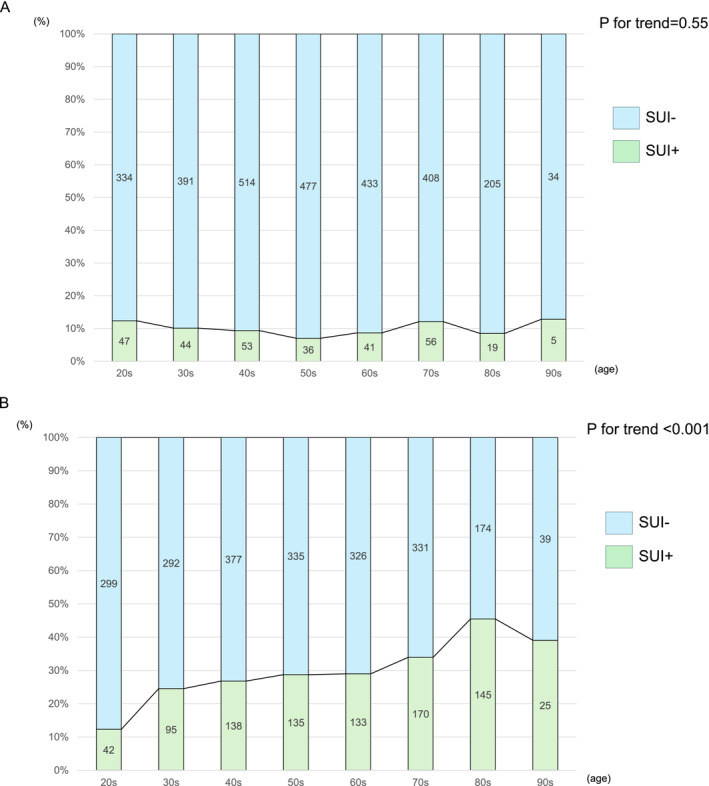
(A) Age distribution of stress urinary incontinence in males. Prevalence of stress urinary incontinence was significantly different between each age group. (B) Age distribution of stress urinary incontinence in females. Prevalence of stress urinary incontinence increased significantly with age. SUI: stress urinary incontinence.

**FIGURE 2:**
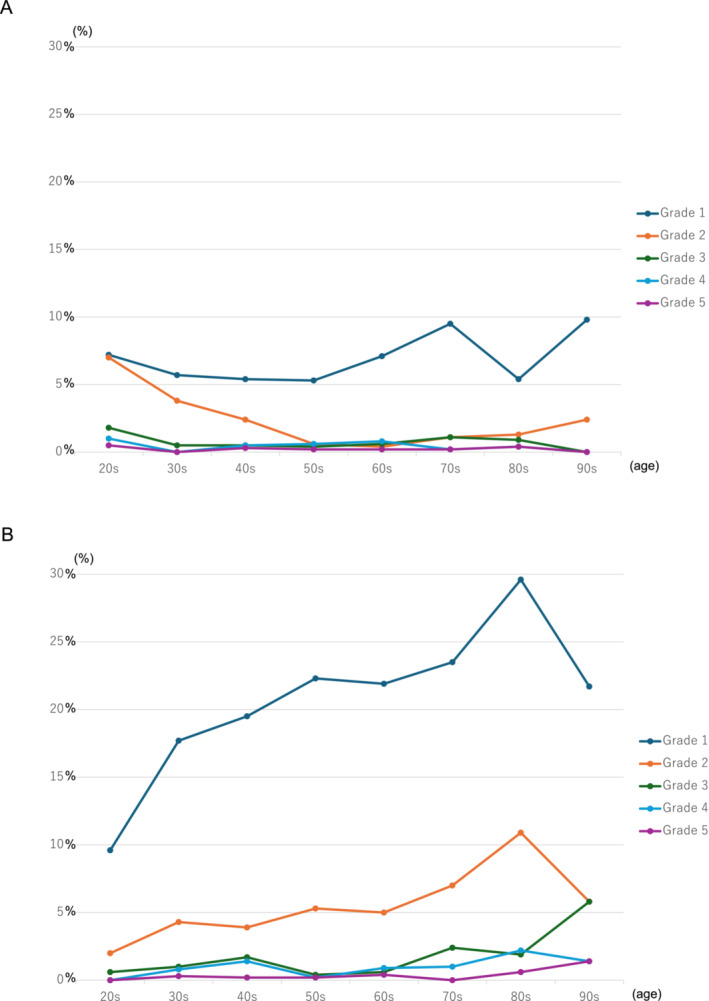
(A) Frequency of stress urinary incontinence in males. Grade 1: stress urinary incontinence (SUI) occurs less often than once per week; Grade 2: SUI occurs once or more than once per week; Grade 3: SUI occurs once per day; Grade 4: SUI occurs several times per day; Grade 5: SUI is always present through the day. (B) Frequency of stress urinary incontinence in females. Grade 1: stress urinary incontinence (SUI) occurs less than once per week; Grade 2: SUI occurs once or more than once per week; Grade 3: SUI occurs once per day; Grade 4: SUI occurs several times per day; Grade 5: SUI occurs through the day.

**FIGURE 3:**
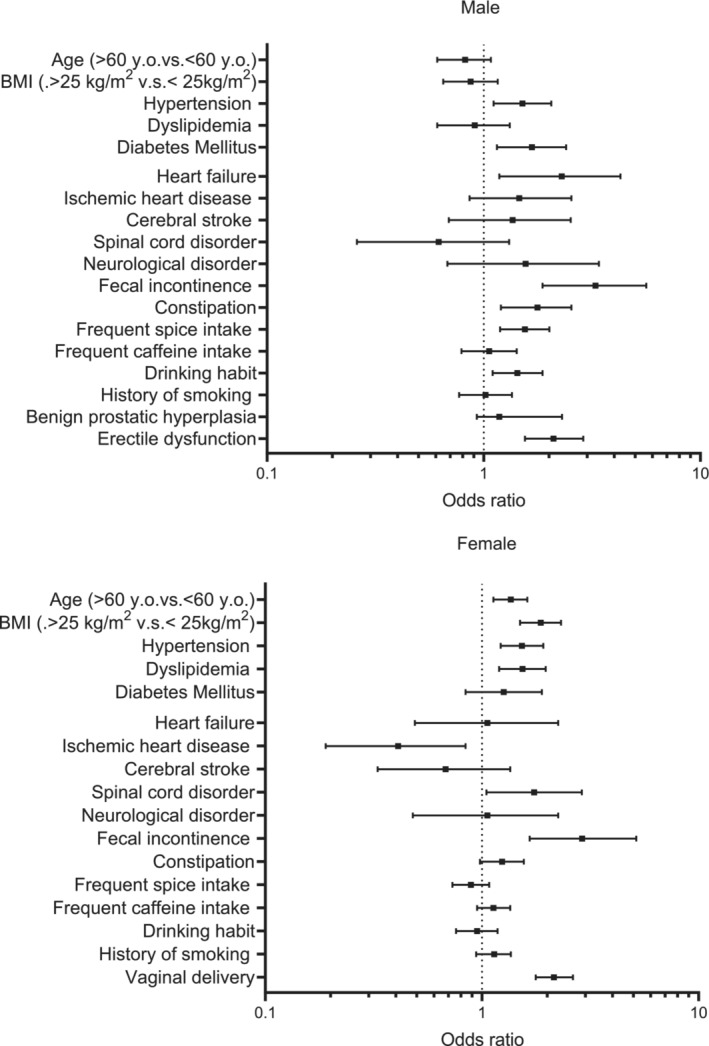
Forest plot of related factors of stress urinary incontinence in both males and females.

The actual number and percentage of participants with SUI among males and females is shown in Figure 1A,B. In males, SUI was observed in about 10% of participants in their 20s or 30s. However, there was no statistically significant difference in the prevalence of SUI with age in males (P = 0.55). Among females, SUI was observed in about 10% of participants in their 20s, which was consistent with the prevalence of SUI in males in their 20s. However, unlike males, the incidence of SUI showed a statistically significant increase with age in females (P < 0.0001). Regarding the frequency of SUI, in both sexes, most participants with SUI had Grade 1 symptoms (Figure 2A,B). In males, the percentage of Grade 1 and 2 SUI gradually decreased with age until their 50s, while in females, the percentage of Grade 1 and 2 SUI gradually increased with age (Figure 3B). Further, in both sexes, only a few subjects had Grade 3 or more SUI.

In multivariate analyses in males, SUI was associated with hypertension (odds ratio (OR), 1.51; 95% confidence interval (CI), 1.11–2.05), diabetes mellitus (OR, 1.67; 95% CI, 1.15–2.40), heart failure (OR, 2.29; 95% CI, 1.18–4.28), faecal incontinence (OR, 3.28; 95% CI, 1.87–5.63), constipation (OR, 1.77; 95% CI, 1.20–2.54), frequent spice intake (OR, 1.55; 95% CI, 1.19–2.01), drinking habit (OR, 1.43; 95% CI, 1.10–1.87) and ED (OR, 2.10; 95% CI, 1.55–2.88) (Figure 3 and Table S1).

In multivariate analyses in females, SUI was significantly associated with age (OR, 1.36; 95% CI, 1.13–1.62), BMI (OR, 1.87; 95% CI, 1.50–2.32), hypertension (OR, 1.53; 95% CI, 1.22–1.92), dyslipidemia (OR, 1.54; 95% CI, 1.20–1.97), spinal cord disorders (OR, 1.74; 95% CI, 1.05–2.89), faecal incontinence (OR, 2.90; 95% CI, 1.66–5.16) and vaginal delivery (OR, 2.15; 95% CI, 1.77–2.63) (Figure 3 and Table S1). In addition, ischemic heart disease (OR, 0.41; 95% CI, 0.19–0.84) had a negative impact on SUI in women (Figure 3 and Table S1).

## 4 DISCUSSION

In the present study, the prevalence and factors associated with SUI were evaluated in more than 6000 participants through a web‐based survey. One of the strongest points of our JaCS study 2023 was that the surveys were conducted in participants aged ≥20 y. Surprisingly, male SUI was observed even in the younger generation, i.e., among those in their 20s and 30s. In the present study, the prevalence of male SUI was about 10%.

The incidence of male SUI was about 3% in the survey conducted 20 years ago,^10^, ^11^ suggesting that the prevalence of male SUI has increased compared with the past survey. On the other hand, another epidemiological study conducted by Abufaraj et al. demonstrated no change in the incidence of female SUI from 2005 to 2018.^20^ The reasons for this discrepancy between the present study and the study by Abufaraj et al. were as follows. First, the definition of SUI was different in the two studies. In our study, SUI was evaluated for one month, while Abufaraj et al. evaluated SUI for one year. Second, health consciousness has increased in Japan. As a result, the participants were sensitive to even short‐term and minor SUIs, which would have been ignored 20 years ago, contributing to the observed increase in SUIs in men compared to the previous data.

In the present survey, while the prevalence of female SUI increased significantly with age, male SUI did not show a significant age‐related change in prevalence. Yet, the frequency of SUI was not very high in either sex. Additionally, the prevalence of mild SUI in males (Grade 1 and 2) decreased from the age of 20s to 50s in the present study. McGrother et al. demonstrated that, in the UK, in men aged more than 40 years, the 1‐year incidence of storage symptoms was 3.8%, with a high remission rate of 39.6%.^21^ This suggests that, in males, mild SUI might naturally improve in subjects aged up to 50 years.

In the present study, the factors related to SUI were investigated from the perspective of participants' characteristics and health‐related behaviours. Age and BMI correlated significantly with only female SUI, which was consistent with previous reports.^22^, ^23^, ^24^ Increase in BMI might induce an increase in abdominal pressure, and ageing might be associated with the weakening of the pelvic floor muscles, both of which would contribute to the increase in female SUI.

Regarding the comorbidities related to SUI, HT was significantly associated with SUI in both sexes in the present study. Markland et al. demonstrated a significant association between HT and UI in men in the 2005–2008 NHANES study.^1^ The authors speculated that medications for HT might affect intravascular volume status, potentially leading to UI.^1^ In the present study, diabetes mellitus and heart failure were significantly associated with only male SUI. The probable reason for the association between diabetes mellitus and SUI could be that diabetes might damage the nerves of the sphincter and/or bladder, leading to damage to the lower urinary tract, and subsequently, SUI.^25^ The association between SUI and heart failure could be due to the use of diuretics, as Poole et al. demonstrated that SUI was more common in patients who used diuretics for heart failure.^26^ However, while several studies demonstrated that diabetes mellitus and heart failure are also significantly associated with SUI in women,^27^, ^28^, ^29^, ^30^ the reasons why these factors were not associated with female SUI in the present study are unknown.

On the other hand, dyslipidemia was significantly associated with female SUI. Dyslipidemia is reportedly exacerbated by the attenuation of oestrogen.^31^ Additionally, attenuation of oestrogen might lead to weakening of the sealing effect of the urethral mucosa.^6^ Thus, a significant association between dyslipidemia and female SUI might result from the attenuation of oestrogen for various reasons. Neurological disorders were also significantly associated with only female SUI. Pelvic nerve damage due to neural disorders can damage the lower urinary tract.^6^ The short urethra in females compared to the long functional urethral length in males might explain why neurological disorders more easily induce SUI in females than males.

In the present study, vaginal delivery was significantly associated with SUI in women. This finding was consistent with the report by Rortveit et al. who demonstrated that women who have experienced vaginal delivery have a significantly higher risk of SUI than women who have not experienced vaginal deliveries (either because they are nulliparous or because they have undergone caesarean sections).^32^ However, although we only investigated the association between vaginal delivery and SUI in the women in the present study, it is possible that the number of pregnancies would influence the frequency and severity of SUI in women. Thus, future studies are needed to clarify this association.

The odds ratio of faecal incontinence was highest in both sexes. In a study conducted in rural China, Luo et al. demonstrated that faecal incontinence was significantly associated with urinary incontinence.^33^ This result was consistent with those of our study. The factors contributing to the simultaneous onset of SUI and faecal incontinence are considered to be congenital or age‐related weakness of pelvic floor muscles and the anal and urethral sphincter, possibly due to occult pudendal neuropathy, damage to the pelvic floor muscles following intrapelvic surgery, vaginal delivery, etc.^34^ Additionally, constipation was significantly associated with only male SUI. Although the occurrence of faecal incontinence and constipation seem contradictory to each other, sarcopenia could induce both types of stool conditions, as well as SUI.^35^ Future studies are needed to clarify the correlations between sarcopenia, stool conditions and SUI.

Regarding the association between health‐related behaviours and SUI, drinking habit and frequent spice consumption were significantly associated with only male SUI. A previous study demonstrated a significant association between female UI and alcohol intake.^36^ Consumption of large amounts of alcohol was associated with a significant increase in the number of participants with male SUI in the present study (data not shown). Thus, intoxication due to alcohol consumption might be involved in male SUI.

To the best of our knowledge, this is the first report demonstrating the association between male SUI and frequent spice consumption. Although the reason for this significant association is unclear, since the high intake of chilli was previously shown to be significantly positively associated with cognitive decline,^37^ some cognitive decline might have triggered male SUI in this study. However, the influence of spice intake on male SUI was not accelerated by ageing in this study (data not shown). Since alcohol consumption and frequent spice intake are controllable factors, these factors are adjustable factors for SUI.

ED was another factor found to be significantly related to male SUI. Serikaya et al. reported that urinary incontinence induces sexual dysfunction.^38^ Thus, in the present study, ED might have been induced as a result of SUI. Another factor potentially contributing to SUI could be that endothelial dysfunction induced by high glucose levels results in microvascular damage, which might subsequently lead to the development of ED, as was reported by Lee et al.^39^ In the present study, a significant association between SUI and diabetes mellitus (DM) and ED was observed. It was, therefore, proposed that systemic microvascular damage is the common underlying factor in DM, ED and SUI. We postulated that microvascular damage to the lower urinary tract, particularly to the internal and external urethral sphincters, might precipitate dysfunction of the lower urinary tract, thereby leading to SUI.

Our study presents the following novel information, based on which we suggest some testable hypotheses. The present study employed a web‐based survey of men and women aged 20 years and older. To date, the majority of large epidemiological studies on SUI have been conducted primarily in women,^40^, ^41^, ^42^ with relatively few studies focusing on men. The majority of studies on SUI in men have been conducted on patients who have undergone surgery for prostate cancer or benign prostatic hyperplasia.^43^, ^44^ However, the present study was conducted to elucidate the prevalence of SUI in a cohort of predominantly male individuals in the general population, including some prostate cancer patients. This study involved determining the prevalence and severity of SUI. It is noteworthy that despite the predominantly healthy cohort, an SUI incidence rate of approximately 10% was identified, particularly in men aged 20 years and above. It is also noteworthy that the frequency of SUI in women increased with age, while no such change was observed in men, regardless of age. This discrepancy might be attributable to anatomical variations in the pelvic cavity, which might contribute to the development of SUI.

It is also noteworthy that the study revealed a correlation between lifestyle‐related factors, such as spice intake and alcohol consumption, and SUI. Moreover, the study revealed an association between male SUI and DM and ED, which are known to be related to vascular endothelial damage.

In light of the above, the following testable hypothesis can be formulated: if spice consumption and alcohol consumption are associated with SUI in men, it would be beneficial to ascertain whether reducing spice and alcohol consumption can be used as a means of improving SUI in the future. Moreover, it is possible that vascular endothelial damage might be a contributing factor to SUI in men. Given that vascular endothelial damage has been demonstrated to reduce growth arrest‐specific protein 6 (Gas6) in plasma,^39^ it might be feasible to ascertain whether vascular endothelial damage is indeed the underlying cause of SUI in men by examining the correlation between SUI and Gas6 in this population.

Several limitations must be considered in the present study. First, because this survey was conducted during the warm season, LUTS might have been mild compared to during cold seasons. Second, since this survey was conducted via web‐based research, there were fewer elderly people in the present study. It is also likely to have included subjects with a higher intellectual level, who might be more familiar with using the internet than the general elderly population. Third, SUI was diagnosed using a self‐reported questionnaire, which might have led to misclassifications. For example, some participants, especially young males, might have misclassified postmicturition dribble as SUI. However, no correlation was found between the frequency of SUI and that of postmicturition dribble (data not shown). Fourth, it should be noted that the questionnaire in the present study did not include a question about whether or not the participants had received treatment for SUI. Therefore, it was not possible to make any adjustments for women who had undergone treatment for their SUI. Hence, it is possible that the frequency and severity of SUI in the study cohort might have been underestimated to some extent because of the possible inclusion of women who had received treatment for SUI. Fifth, it is also possible that postmicturition dribble in males was misinterpreted as SUI, although the present study did not observe a correlation between the frequency of SUI and the frequency of postmicturition dribble (data not shown). Sixth, we did not validate the association between actual SUI and the subjects' answers regarding the presence or absence of SUI. Finally, since this was a cross‐sectional design study, causality between SUI and its related factors could not be determined.

## 5 CONCLUSIONS

In this nationwide web‐based survey in Japan, the frequency of SUI was low in both sexes. However, the trend between the prevalence of SUI and age was different between both sexes. The prevalence of male SUI was consistently about 10% through all age groups, while that of female SUI increased with age. Especially in males, mild SUI improved with age until the 50s. Female SUI might involve weakness of the pelvic floor muscles, while male SUI might be associated with health‐related behaviours.

## AUTHOR CONTRIBUTIONS

Nobuhiro Haga, Mikako Yoshida and Takahiko Mitsui designed the research. Noritohi Sekido and Naoya Masumori analysed data. Kenji Omae and Motoaki Saito prepared the manuscript. Yasue Kubota and Ryuji Sakakibara helped with the research and writing the manuscript. Satoru Takahashi supervised the study. All authors reviewed the manuscript.

## CONFLICT OF INTEREST STATEMENT

Takahiko Mitsui declares support from The 50th Anniversary Project of the Japanese Continence Society and payments from Astellas Pharma Inc., KYORIN Pharmaceutical Co., Ltd. and Kissei Pharmaceutical Co., Ltd as well as payment for expert testimony from KYORIN Pharmaceutical Co., Ltd. All other authors declare no conflict of interest.

## Supporting information


**Table S1.** Logistic analyses for stress urinary incontinence both in male and female.
