# Supplementary material for: Prevalence and risk factors of stress urinary incontinence in a 2023 Japanese community health survey ‐ differences between males and females
Source: BJUI Compass. 2025 Feb 17;6(2):e70004. doi: 10.1002/bco2.70004 (PMC11830994; doi:10.1002/bco2.70004)
Supplement: Supplementary file 1 — Table S1. Logistic analyses for stress urinary incontinence both in male and female. [file BCO2-6-e70004-s001.pptx]

## Slide 1
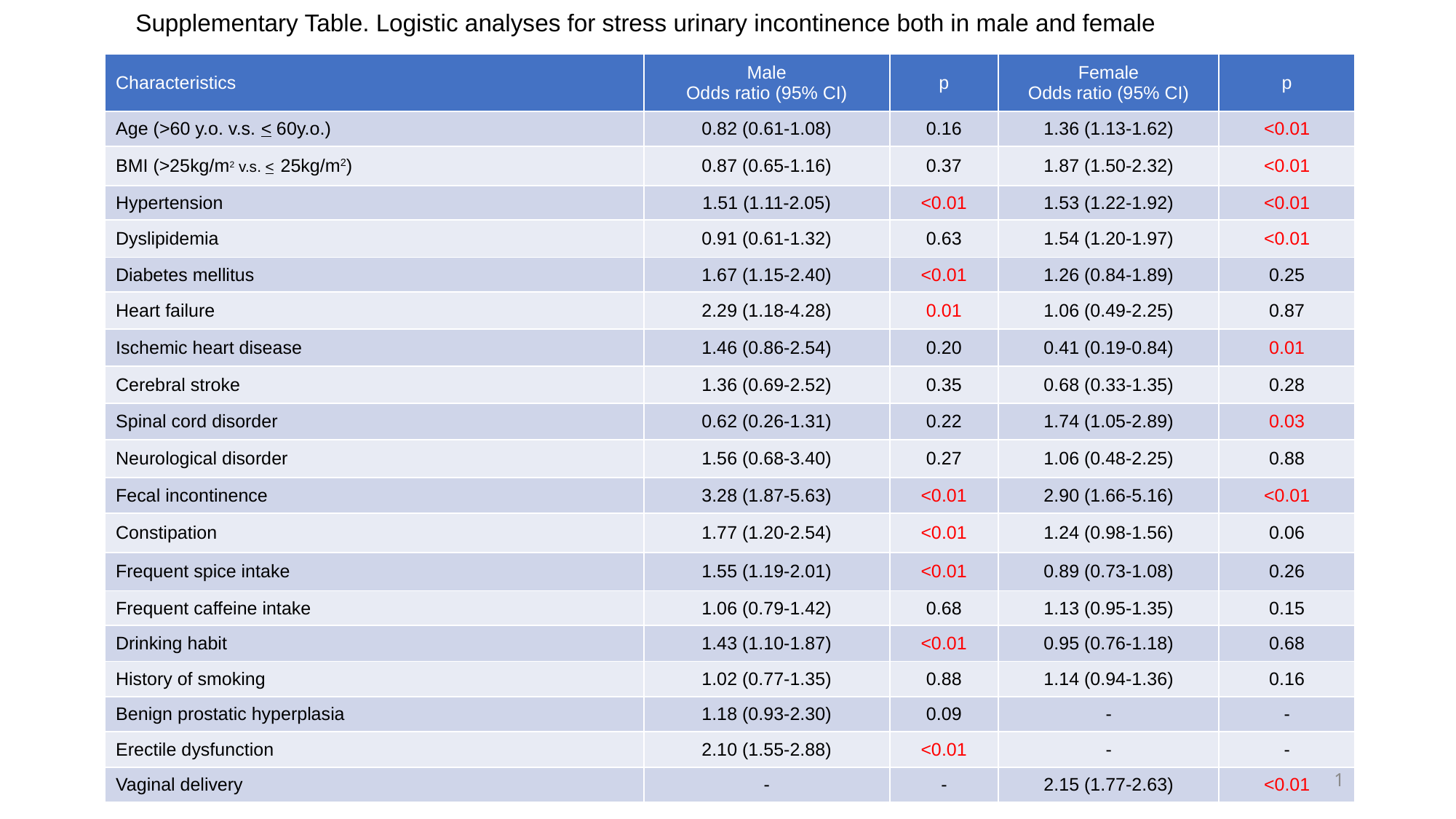

Supplementary Table. Logistic analyses for stress urinary incontinence both in male and female
| Characteristics | Male Odds ratio (95% CI) | p | Female Odds ratio (95% CI) | p |
| --- | --- | --- | --- | --- |
| Age (>60 y.o. v.s. < 60y.o.) | 0.82 (0.61-1.08) | 0.16 | 1.36 (1.13-1.62) | <0.01 |
| BMI (>25kg/m2 v.s. < 25kg/m2) | 0.87 (0.65-1.16) | 0.37 | 1.87 (1.50-2.32) | <0.01 |
| Hypertension | 1.51 (1.11-2.05) | <0.01 | 1.53 (1.22-1.92) | <0.01 |
| Dyslipidemia | 0.91 (0.61-1.32) | 0.63 | 1.54 (1.20-1.97) | <0.01 |
| Diabetes mellitus | 1.67 (1.15-2.40) | <0.01 | 1.26 (0.84-1.89) | 0.25 |
| Heart failure | 2.29 (1.18-4.28) | 0.01 | 1.06 (0.49-2.25) | 0.87 |
| Ischemic heart disease | 1.46 (0.86-2.54) | 0.20 | 0.41 (0.19-0.84) | 0.01 |
| Cerebral stroke | 1.36 (0.69-2.52) | 0.35 | 0.68 (0.33-1.35) | 0.28 |
| Spinal cord disorder | 0.62 (0.26-1.31) | 0.22 | 1.74 (1.05-2.89) | 0.03 |
| Neurological disorder | 1.56 (0.68-3.40) | 0.27 | 1.06 (0.48-2.25) | 0.88 |
| Fecal incontinence | 3.28 (1.87-5.63) | <0.01 | 2.90 (1.66-5.16) | <0.01 |
| Constipation | 1.77 (1.20-2.54) | <0.01 | 1.24 (0.98-1.56) | 0.06 |
| Frequent spice intake | 1.55 (1.19-2.01) | <0.01 | 0.89 (0.73-1.08) | 0.26 |
| Frequent caffeine intake | 1.06 (0.79-1.42) | 0.68 | 1.13 (0.95-1.35) | 0.15 |
| Drinking habit | 1.43 (1.10-1.87) | <0.01 | 0.95 (0.76-1.18) | 0.68 |
| History of smoking | 1.02 (0.77-1.35) | 0.88 | 1.14 (0.94-1.36) | 0.16 |
| Benign prostatic hyperplasia | 1.18 (0.93-2.30) | 0.09 | - | - |
| Erectile dysfunction | 2.10 (1.55-2.88) | <0.01 | - | - |
| Vaginal delivery | - | - | 2.15 (1.77-2.63) | <0.01 |
1
